# Cyanobacteria-Derived Extracellular Vesicles: A Novel Frontier in Drug Delivery and Therapeutics

**DOI:** 10.3390/ijms27010004

**Published:** 2025-12-19

**Authors:** Khalid A. Asseri, Krishnaraju Venkatesan, Yahya I. Asiri, Saud Alqahtani, Taha Alqahtani, Pooja Muralidharan, Shaimaa Elsayed Ramadan Genena, Durgaramani Sivadasan, Premalatha Paulsamy, Kumarappan Chidambaram

**Affiliations:** 1 Department of Pharmacology, College of Pharmacy, King Khalid University, Abha 61421, Saudi Arabia; 2 Undergraduate Program, PSG College of Pharmacy, Peelamedu, Coimbatore 641004, India; 3 Clinical Biochemistry Department, University of Tabuk, Tabuk 41477, Saudi Arabia; 4 Department of Pharmaceutics, College of Pharmacy, Jazan University, Jizan 45142, Saudi Arabia; 5 College of Nursing, Mahalah Branch for Girls, King Khalid University, Khamis Mushayt 61421, Saudi Arabia; 6 Department of Pharmacology and Toxicology, College of Pharmacy, King Khalid University, Abha 61421, Saudi Arabia

**Keywords:** cyanobacterial EVs, drug delivery systems, bioactive compounds, targeted therapeutics, nanocarriers

## Abstract

Cyanobacteria, known for their diverse and potent bioactive compounds, present a unique method for drug delivery via their extracellular vesicles (EVs), often described as exosome-like due to size and function but distinct in biogenesis. These naturally occurring vesicles, particularly those from cyanobacteria, are gaining attention as potential carriers for targeted drug delivery because of their biocompatibility, stability, and ability to encapsulate various bioactive compounds. However, cyanobacterial EVs remain underexplored as a dedicated nanocarrier platform, and their specific advantages and limitations relative to existing systems have not been systematically synthesized. This review explores the potential therapeutic uses of cyanobacterial EVs, emphasizing their roles in cancer treatment, antimicrobial therapies, neuroprotection, and immune modulation. We explore their biogenesis and structural features, comparing them to synthetic nanocarriers like polymeric nanoparticles and liposomes. The review also addresses the challenges of isolating and characterizing cyanobacterial EVs at scale and highlights the need for advancements in synthetic biology and genetic engineering to optimize their therapeutic potential. Despite these challenges, cyanobacterial EVs’ unique properties offer significant promise for advancing drug delivery systems and providing innovative solutions for treating complex diseases.

## 1. Introduction

Cyanobacteria, or blue-green algae, are among the most ancient organisms on Earth, with a history spanning over 2.5 billion years. These photosynthetic bacteria have played a critical role in shaping Earth’s atmosphere, particularly by contributing to the production of oxygen. Due to their unique metabolic capabilities, cyanobacteria have emerged as valuable candidates in various biotechnological applications [1]. A key example is *Synechocystis* sp. PCC6803, the first photosynthetic organism to have its genome fully sequenced, which has served as a model for genetic and metabolic studies. Such advancements have opened doors for engineering cyanobacteria to produce biofuels, such as ethanol and hydrogen, providing sustainable alternatives to fossil fuels. Furthermore, modifications to the Calvin-Benson-Bassham (CBB) cycle have demonstrated an enhancement in ethanol production in these organisms [2,3]. Beyond energy production, cyanobacteria are being explored for their ability to synthesize bioactive compounds with therapeutic properties, such as antimicrobial and anti-inflammatory substances, thereby positioning them as potential resources in pharmaceutical development [4].

In addition to their biotechnological potential, cyanobacteria contribute to environmental sustainability. Their ability to fix atmospheric nitrogen and sequester carbon dioxide positions them as biofertilizers, enhancing soil quality and supporting plant growth. Moreover, cyanobacteria-based biofilms have been utilized in wastewater treatment to remove nutrients and heavy metals, highlighting their versatility in addressing pressing environmental challenges [5]. Given their adaptability and diverse metabolic pathways, cyanobacteria continue to inspire innovative applications across a range of sectors, reinforcing their significant biotechnological potential.

An emerging yet underexplored area of research involves the study of extracellular vesicles (EVs), particularly exosome-like vesicles (ELVs) derived from cyanobacteria, as a novel platform for drug delivery. EVs are membrane-bound particles secreted by cells into the extracellular space, playing a key role in cell communication and the transfer of bioactive molecules. Exosomes, a specific type of EV, are small vesicles ranging from 30 to 150 nanometers in diameter, derived from the endosomal pathway. These vesicles are vital for intercellular communication, carrying a wide range of molecules, including lipids, proteins, and nucleic acids, that can affect recipient cells and regulate various biological processes such as angiogenesis, tumor progression, and immune responses [6,7]. Although exosomes have been widely studied in mammalian systems, EVs in microbial species such as cyanobacteria remain a promising and evolving field of study.

Drug delivery systems based on natural products represent another promising area of therapeutic advancement. These systems leverage the inherent properties of biological materials to improve therapeutic outcomes by offering superior biocompatibility, reduced toxicity, and favorable interactions with biological tissues [8]. For example, polysaccharides like pullulan and natural polymers like alginate have been employed in targeted cancer therapies and drug release systems, enabling controlled drug release and minimizing adverse effects on healthy tissues [9]. The use of DNA nanostructures for precise drug delivery further highlights the potential of naturally derived systems in enhancing specificity and reducing systemic toxicity [10].

“Cyanobacteria-derived EVs (including exosome-like vesicles) represent a promising but emerging convergence of natural product therapeutics and biological nanocarriers. Cyanobacterial EVs have been shown to be stable, biocompatible, and able to carry biomolecules. For instance, *Spirulina*-derived EVs have been isolated and characterized, demonstrating potential for adjuvant use [11]. Furthermore, vesicles from photosynthetic microalgae have been engineered to deliver therapeutic proteins to mammalian cells [12]. Reviews on cyanobacterial EVs highlight their ecological roles and suggest potential for biotechnological exploitation [8]. However, direct evidence for the encapsulation and delivery of endogenous cyanobacterial metabolites (e.g., anticancer, antimicrobial) in therapeutic settings remains limited and requires further research.”

While numerous reviews have examined cyanobacterial secondary metabolites or exosomes in general, a focused synthesis that positions cyanobacterial EVs within the broader nanocarrier landscape is still absent [13,14]. As a result, the specific advantages and translational challenges of cyanobacterial EV-based drug delivery remain poorly defined. Nanocarrier research has advanced rapidly, with liposomes, polymeric nanoparticles, and mammalian exosomes already evaluated in preclinical and clinical settings [15,16]. However, synthetic nanocarriers often require complex chemistries, may elicit off-target toxicity or rapid clearance, and are expensive to produce at scale [17]. Mammalian exosomes, although biologically sophisticated, are limited by donor variability, scalability constraints, and stringent biosafety and ethical requirements [18]. Microalgal vesicles provide a more sustainable option, yet they are typically treated as generic plant- or algal-derived vesicles rather than as species-tailored, metabolite-enriched carriers [12].

Cyanobacteria occupy a distinctive niche in this landscape. They are photosynthetic, fast-growing prokaryotes that can be cultivated at large scale in simple media and are amenable to genetic engineering [1,2]. Many cyanobacterial species naturally synthesize highly potent anticancer, antimicrobial, antiviral, and immunomodulatory metabolites that could, in principle, be selectively packaged into their EVs. Cyanobacterial EVs therefore offer a unique combination of (i) sustainable, light-driven production, (ii) intrinsically bioactive cargo, and (iii) compatibility with microbial synthetic biology for programmable vesicle biogenesis and surface display [13,19].

Therefore, cyanobacterial EVs represent a biologically compatible, environmentally sustainable, and potentially customizable drug delivery system that could address current limitations in synthetic nanocarriers. With their unique combination of biocompatibility, low toxicity, and ecological adaptability, cyanobacterial EVs present a compelling platform for the development of next-generation therapeutic delivery systems [8].

This review aims to provide a comprehensive and critical overview of the emerging field of cyanobacterial EVs, highlighting their biogenesis, therapeutic potential, advantages over conventional systems, and future opportunities for bioengineering and clinical application. Specifically, it (1) summarizes current knowledge on cyanobacterial EV biogenesis, composition, and inter-kingdom communication; (2) integrates these data with the pharmacology of cyanobacterial metabolites to propose therapeutically relevant payloads; (3) compares cyanobacterial EVs with conventional synthetic and biological nanocarriers; and (4) outlines key technological, regulatory, and safety challenges that must be resolved to translate cyanobacterial EVs into clinically meaningful drug-delivery systems.

## 2. Cyanobacteria as a Source of Natural Bioactive Compounds

### 2.1. Overview of Bioactive Metabolites in Cyanobacteria

Cyanobacteria are a varied group of microorganisms recognized for their extensive production of bioactive metabolites, including peptides, alkaloids, terpenes, and polyketides, many of which exhibit powerful pharmacological effects such as anticancer, antimicrobial, antiviral, and UV-protective properties. These secondary metabolites demonstrate a broad spectrum of biological activities, making cyanobacteria a valuable resource for pharmaceutical and industrial applications. More than 2000 secondary metabolites have been identified in cyanobacteria, highlighting their vast potential for drug discovery and development [20].

Among the peptides produced by cyanobacteria, microcystins, nodularins, cylindrospermopsin, and cyanopeptolins stand out for their strong ability to inhibit specific protein phosphatases and serine proteases. Although these compounds may pose health risks due to their toxicity, they also offer considerable potential for creating targeted therapies with fewer side effects than conventional drugs [21,22]. These peptides have also contributed to advancements in cancer biology and cell signaling. In addition to peptides, cyanobacteria produce alkaloids, such as saxitoxin and anatoxin-a, which are known for their neurotoxic effects. Despite their toxicity, these compounds are of great interest in drug development, particularly for targeting neural pathways, with their unique chemical structures offering valuable insights for designing new therapeutics [23].

Cyanobacteria also generate polyketides like curacin A and apratoxins, which exhibit significant cytotoxic properties, making them promising candidates for cancer treatment. These compounds interfere with microtubule assembly, a critical process in cell division, which is a key target in cancer chemotherapy [19]. Furthermore, terpenes and lipid-derived metabolites contribute additional chemical diversity. These compounds have demonstrated various bioactivities, including antimicrobial, antifungal, and anticancer properties, underscoring both the ecological significance and pharmaceutical applications of cyanobacterial metabolites [13].

### 2.2. Comparison with Other Microalgae-Derived Natural Products

Cyanobacteria, together with other microalgae like green algae and diatoms, are abundant sources of bioactive compounds that hold considerable promise for nutraceutical, pharmaceutical, and industrial uses. While both groups share certain capabilities, they also show distinct differences in their metabolite profiles and primary applications.

Cyanobacteria are particularly known for synthesizing a diverse range of secondary metabolites such as alkaloids, peptides, and polyketides which exhibit a wide spectrum of biological activities, including anticancer, antimicrobial, antiviral, and antifungal properties. For example, compounds like nodularins and microcystins are potent inhibitors of protein phosphatases, while curacin A and apratoxins have demonstrated significant cytotoxic properties against cancer cells [24].

In contrast, other microalgae such as green algae and diatoms primarily produce metabolites like lipids, carotenoids, and polysaccharides. These compounds are highly valued for their applications in industries such as biofuels, nutraceuticals, and natural colorants. For instance, microalgal lipids are explored for biofuel production due to their high oil content, while carotenoids such as β-carotene and astaxanthin are utilized as dietary supplements and natural pigments [25].

Both cyanobacteria and other microalgae have been recognized for their anticancer properties. Recent studies have highlighted that bioactive substances from these organisms, including tannins, phenolic acids, terpenoids, and flavonoids, exhibit anticancer effects by inducing apoptosis, inhibiting telomerase, modulating protein kinases, and influencing epigenetic modifications [14]. However, cyanobacteria are distinguished by their broader chemical diversity and stronger cytotoxicity profiles, which are particularly relevant for therapeutic applications requiring precise intracellular targeting. Integrating these high-value compounds into ELVs could further enhance their therapeutic potential by improving delivery specificity, stability, and cellular uptake [26].

### 2.3. Potential for Therapeutic Applications

Cyanobacteria-derived metabolites have demonstrated significant therapeutic potential in various areas, including anticancer, antimicrobial, anti-inflammatory, hepatoprotective, and neuroprotective effects. The antimicrobial activity of these compounds is among the most well-studied. For example, certain cyanobacterial metabolites, such as berberine, have shown the ability to inhibit bacterial quorum sensing a critical system that regulates bacterial virulence and biofilm formation. Additionally, lipid compounds from cyanobacteria disrupt bacterial cell membranes, leading to cell lysis and inhibition of key bacterial pathways such as the Shikimate pathway [27]. These mechanisms position cyanobacteria as a valuable source of novel antimicrobial agents.

The anticancer potential of cyanobacterial compounds has also garnered considerable attention. Compounds like phycocyanin, a pigment-protein complex derived from *Spirulina* (*Arthrospira*), have been shown to halt the cell cycle prior to the S phase and induce apoptosis through the generation of reactive oxygen species (ROS). This activity leads to reduced BCl-2 expression and increased caspase activation [28,29]. While phycocyanin alone may not achieve complete tumor regression, its encapsulation into delivery systems such as exosomes may enhance its bioavailability and therapeutic impact in combination therapies.

Cyanobacteria also offer anti-inflammatory and antioxidant benefits, particularly through compounds like phycocyanin, which has been found to scavenge peroxyl, hydroxyl, and alkoxyl radicals. This activity reduces oxidative stress and inflammation, making it a potential agent for treating conditions such as rheumatoid arthritis and cardiovascular diseases [30]. Furthermore, cyanobacterial metabolites exhibit neuroprotective effects. Phycocyanin, for instance, scavenges hydrogen peroxide in astrocytes, reducing neuroinflammation and oxidative stress. It also enhances the production of growth factors such as BDNF and NDF (Neurotrophic Differentiation Factor), promoting neuronal regeneration and preventing astrogliosis hallmarks of neurodegenerative diseases like Parkinson’s and Alzheimer’s [30].

Finally, phycocyanin has shown hepatoprotective properties by interfering with the cytochrome P-450 system, thereby preventing the formation of toxic intermediates and mitigating oxidative liver damage [30]. Taken together, these therapeutic attributes make cyanobacterial compounds strong candidates for incorporation into next-generation drug delivery systems. Their bioactive potential, when combined with targeted delivery via exosomes, could enable precise intervention in complex disease pathways with minimal off-target effects [26]. As summarized in Table 1, several key metabolites from cyanobacteria exhibit strong therapeutic activities across cancer, microbial infections, and neuroinflammation. Their encapsulation within ELVs may enhance target specificity, reduce systemic toxicity, and support innovative drug delivery platforms.

**Figure 1:**
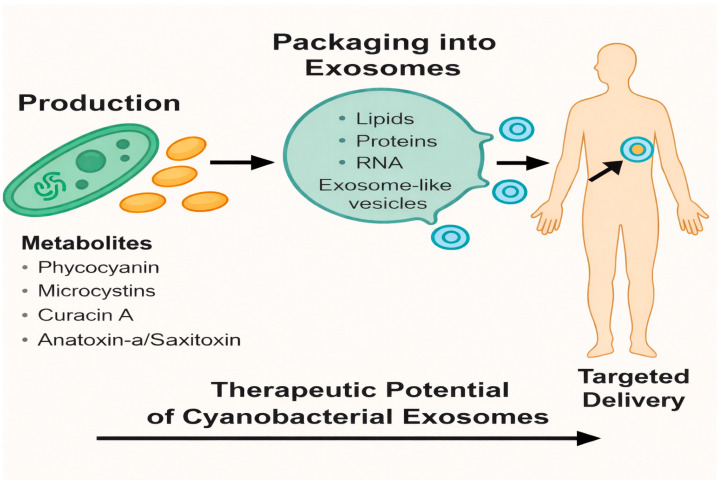
Schematic Representation of the Therapeutic Potential of Cyanobacterial EVs. Cyanobacteria synthesize a wide range of bioactive metabolites, including phycocyanin, microcystins, curacin A, and anatoxin-a/saxitoxin. These metabolites can be naturally or artificially encapsulated into ELVs composed of lipids, proteins, and nucleic acids. Once packaged, these vesicles can serve as biologically compatible nanocarriers for targeted delivery to diseased tissues, offering promising therapeutic applications in cancer, neurodegeneration, and infectious diseases.

Figure 1 presents an overview of how cyanobacterial EV-like vesicles serve as promising platforms for therapeutic delivery. Cyanobacteria produce a wide spectrum of biologically active metabolites with known pharmacological properties. These compounds can be selectively packaged into exosome-like vesicles enriched with functional biomolecules including lipids, proteins, and RNA. Once released, these vesicles exhibit the ability to interact with human cells and facilitate targeted delivery of their encapsulated components. The integration of cyanobacterial metabolites into natural vesicle-based carriers highlights a novel strategy for exploiting microbial bioactive in precision medicine, demonstrating significant potential for therapeutic development.

## 3. Exosomes and Their Role in Drug Delivery

### 3.1. Structure, Function, and Biogenesis of Exosomes

In eukaryotic cells, exosomes are small EVs, generally ranging from 30 to 150 nanometers in diameter, enclosed within a lipid bilayer membrane. They are secreted by various cell types into bodily fluids such as blood, urine, saliva, and cerebrospinal fluid and serve as mediators of intercellular communication. Exosomes are enriched with specific lipids, phosphatidylserine, cholesterol, and sphingomyelin that maintain their structural integrity. Their surface typically expresses tetraspanins (CD9, CD81, CD63), ESCRT components (TSG101, Alix), fusion proteins (annexins, flotillins, GTPases), and heat shock proteins (Hsp70, Hsp90), which reflect their endosomal origin. Exosomal cargo also includes nucleic acids (mRNA, miRNA, lncRNA, and occasionally DNA), which can modulate gene expression and function in recipient cells [15].

Table 2 outlines the essential structural and functional attributes of exosomes. The formation of exosomes begins with the inward budding of the plasma membrane to generate early endosomes, which mature into late endosomes. Further invagination within these structures forms intraluminal vesicles (ILVs), resulting in multivesicular bodies (MVBs). MVBs can either fuse with lysosomes for degradation or with the plasma membrane, releasing ILVs as exosomes into the extracellular space. This biogenesis is primarily governed by the endosomal sorting complex required for transport (ESCRT) pathway (ESCRT-0 to ESCRT-III), responsible for membrane budding and cargo sorting. ESCRT-independent pathways, involving tetraspanins, ceramides, and lipid rafts, also contribute to exosome formation [16].

In contrast, prokaryotic cells, including cyanobacteria, do not possess an endosomal–MVB system and therefore do not generate exosomes in the strict sense. Instead, their extracellular vesicles typically correspond to outer membrane vesicles (OMVs), which arise by outward blebbing and pinching off of the outer membrane, often encapsulating portions of the periplasmic space [38,39]. These OMVs can overlap with exosomes in size and general function (e.g., cargo transport and intercellular signaling) but are fundamentally distinct in their biogenesis and molecular markers. Throughout this review, vesicles released by cyanobacteria are therefore referred to as extracellular vesicles (EVs) or OMV-like/exosome-like vesicles, to reflect this prokaryotic mode of origin while acknowledging their functional analogy to eukaryotic exosomes [8,38].

**Table 2:** Key Structural and Functional Features of Exosomes.

Feature	Description	References
Origin	Endosomal pathway in eukaryotic cells	[40]
Size	40–160 nm	[18]
Surface markers	CD9, CD63, CD81, Hsp70, Hsp90, MHC, TSG101, ALIX	[41]
Biofluid presence	Found in blood, saliva, urine, cerebrospinal fluid	[42]
Biogenesis process	Endocytosis → ILV formation → MVB maturation → Fusion with plasma membrane	[40]
Pathways	ESCRT-dependent and ESCRT-independent (tetraspanins, ceramides)	[43]

Exosomes play essential roles in immune regulation, cellular signaling, and antigen presentation. Through transfer of lipids, proteins, and RNAs, they reprogram recipient cells, influencing both physiological and pathological conditions. Because their cargo reflects the cellular state of origin, exosomes are being widely investigated as biomarkers and therapeutic nanocarriers [44].

### 3.2. Unique Advantages of Exosome-Based Drug Delivery Systems

Exosome-based drug delivery systems offer distinct advantages over conventional synthetic nanocarriers due to their biological origin, natural compatibility, and targeting capabilities. Their low immunogenicity and biocompatibility stem from their derivation from endogenous cells, minimizing immune responses and enhancing safety profiles [43].

One of the most compelling features of exosomes is their ability to cross biological barriers, including the blood-brain barrier (BBB), thereby allowing access to previously inaccessible tissues [45,46]. Their membrane-bound structure protects encapsulated therapeutic cargos, such as RNA, proteins, or small molecules from enzymatic degradation, enabling increased circulation time and sustained drug release.

Exosomes are naturally taken up by recipient cells via endocytosis, phagocytosis, or receptor-mediated mechanisms, ensuring efficient intracellular delivery. Their surface markers and lipid composition confer cell-type-specific targeting, reducing off-target effects and systemic toxicity [47]. In the context of cyanobacteria, ELVs may possess similar surface properties that facilitate uptake by mammalian cells, though this remains to be fully explored [8]. If validated, these vesicles could serve as biologically sustainable delivery vehicles for antimicrobial peptides, metabolic inhibitors, or immunomodulators. However, their uptake mechanisms, half-life, and circulation behavior require detailed study.

### 3.3. Comparison with Synthetic Nanocarriers

Exosomes offer several functional advantages over conventional synthetic nanocarriers such as liposomes and polymeric nanoparticles, though each system presents unique trade-offs. Liposomes, which are artificially engineered vesicles composed of phospholipid bilayers, are widely used for delivering both hydrophilic and hydrophobic drugs. However, they are often hampered by limited stability in systemic circulation, which may lead to premature drug release and a consequent reduction in therapeutic efficacy. Additionally, liposomes can trigger immune responses depending on their size, surface charge, and lipid composition, thereby limiting their tolerability in certain patient populations [17,48].

Polymeric nanoparticles commonly synthesized from biodegradable materials such as poly(lactic-co-glycolic acid) (PLGA) are another well-established class of drug carriers. They offer high drug-loading capacity and controlled-release profiles, making them attractive for sustained therapeutic delivery. However, their fabrication often requires complex chemical processes, and even with surface modifications, they may not achieve the same degree of biological specificity or cellular targeting as exosomes [49].

In contrast, exosomes inherently possess membrane proteins and lipid components derived from their parent cells, which facilitate natural targeting to specific cell types. This biological origin enhances their compatibility with endogenous cellular processes and improves their uptake efficiency. Moreover, exosomes can cross biological barriers, such as the blood-brain barrier, and evade immune surveillance more effectively than synthetic carriers [50].

Nonetheless, several limitations challenge the clinical translation of exosome-based delivery systems. Large-scale production and purification of exosomes remain technically difficult, often yielding low quantities with significant batch-to-batch variability. Additionally, their loading efficiency, particularly for large macromolecules, can be inferior to that of engineered nanoparticles, which can be readily customized for optimal cargo retention and release [51,52].

Microbial-derived EVs, such as those from cyanobacteria, face further regulatory and technical hurdles. Their bacterial origin raises concerns about the presence of immunostimulatory components like endotoxins and uncharacterized biomolecules, complicating the development of standardized protocols for safety testing. As a result, before microbial EVs can be positioned as viable drug delivery systems, significant advancements are needed in the areas of good manufacturing practice (GMP) compliance, scalable isolation techniques, and biosafety characterization. Addressing these challenges will be critical for harnessing the full therapeutic potential of cyanobacterial vesicles.

## 4. Evidence of ELVs in Cyanobacteria

### 4.1. Reports on Cyanobacterial EVs

A growing body of research has demonstrated that various cyanobacterial species secrete EVs, contributing significantly to both ecological and biomedical functions. These vesicles have been shown to mediate nutrient exchange, respond to stress stimuli, and facilitate intercellular communication within microbial communities. Among the most extensively studied are the marine cyanobacteria *Prochlorococcus* and *Synechococcus*, which produce EVs enriched with diverse biomolecules that participate in horizontal gene transfer, quorum sensing, and ecosystem-level nutrient cycling [38,53].

These vesicles support dynamic communication between microbial cells and their environment, especially within oceanic ecosystems by modulating gene expression, influencing microbial consortia behavior, and facilitating the transport of metabolites. Recent advancements in vesicle isolation from species such as *Prochlorococcus*, *Synechococcus*, and *Synechocystis* have enabled proteomic and lipidomic profiling of cyanobacterial EVs, uncovering their molecular composition and functional capabilities [54].

Notably, EVs from *Spirulina* (*Arthrospira platensis*) have shown biomedical relevance. Their potential as vaccine adjuvants and immunomodulators is gaining attention due to their low toxicity and natural origin, suggesting applicability in oral or topical delivery platforms [11]. Additionally, *Synechococcus elongatus* PCC7942 produces EVs that enhance endothelial cell proliferation and angiogenesis, accelerating wound healing in murine models through enhanced capillary formation and tissue regeneration [55].

These findings suggest that cyanobacterial EVs are not merely passive byproducts but actively participate in adaptive physiology, microbial ecology, and potentially therapeutic signaling. Continued exploration into their cargo specificity, secretion mechanisms, and host interactions could unlock new biotechnological strategies for both environmental and clinical applications.

### 4.2. Key Cyanobacterial Species Producing EVs/Exosome-like Vesicles

Several cyanobacterial species have been extensively studied for their ability to secrete EVs, each contributing uniquely to ecological functions and potential therapeutic applications. Among these, *Prochlorococcus* spp., particularly strains MIT9312 and MIT9313, are among the most abundant photosynthetic organisms in marine ecosystems. These species release EVs that play a central role in nutrient cycling, microbial communication, and the transfer of organic materials within the ocean’s microbial loop. The vesicles carry DNA, proteins, and metabolites, enabling interactions between *Prochlorococcus* and co-occurring microbes, thereby fostering symbiotic relationships and supporting environmental stability [38,56]. Given their nanoscopic size, compositional richness, and environmental resilience, EVs from *Prochlorococcus* have garnered interest for potential applications in marine biotechnology and drug delivery systems adapted to aquatic environments.

Another species of growing biomedical interest is *Synechococcus elongatus* PCC7942, a unicellular cyanobacterium known to secrete EVs with pro-angiogenic properties. These vesicles have demonstrated the ability to promote angiogenesis by enhancing proliferation and migration of human microvascular endothelial cells (HMECs). In preclinical models, such EVs have accelerated wound healing by stimulating new capillary formation, highlighting their promising role in regenerative medicine and tissue repair [39,57].

*Synechocystis* sp. PCC 6803, one of the best-characterized cyanobacterial models, also produces EVs under stress conditions such as oxidative stress or nutrient deprivation. These vesicles are enriched in outer membrane lipids, periplasmic proteins, and small metabolites, and may serve adaptive functions including detoxification, stress response modulation, and intercellular signaling. Their ability to carry biologically active cargo makes them promising candidates for engineering drug delivery platforms [8,58].

*Cylindrospermopsis raciborskii*, a filamentous cyanobacterium frequently associated with harmful algal blooms, is known for producing toxins such as cylindrospermopsin. It also secretes EVs containing proteins, lipids, and possibly toxin-conjugated molecules. While these EVs may contribute to microbial competition and environmental adaptation, their mechanisms remain underexplored. Nonetheless, the presence of structured vesicular secretion in this species presents opportunities for application in environmental biosensing and bioremediation strategies aimed at freshwater ecosystems [39,59].

*Anabaena* sp. PCC 7120, a filamentous, nitrogen-fixing cyanobacterium, has been observed to produce EVs, especially under environmental stress. Although the molecular content and precise function of these vesicles remain to be fully elucidated, it is hypothesized that they may facilitate intra-filament communication or transport nitrogenase-related enzymes. These features suggest a potential role in maintaining nitrogen balance in aquatic ecosystems, and their vesicle system may be adapted for use in biofertilizer technologies [60].

*Spirulina*, also known as *Arthrospira platensis*, is widely recognized for its nutraceutical properties and is one of the few edible cyanobacteria with commercial applications. Recent evidence suggests that *Spirulina* also produces EVs, which may carry bioactive compounds involved in cell-to-cell signaling and immune modulation. These vesicles hold potential in functional foods, vaccine adjuvants, and anti-inflammatory therapeutics [61,62].

In summary, cyanobacterial EVs represent a taxonomically diverse and functionally versatile group of nanostructures. Their roles span ecological maintenance, nutrient recycling, microbial communication, and emerging therapeutic applications. The continued exploration of species-specific EV composition, secretion triggers, and delivery capacity is essential for realizing their full biotechnological and pharmaceutical potential. As summarized in Table 3, diverse cyanobacterial species secrete EVs with distinct compositions and biological roles. Their potential applications span marine biotechnology, drug delivery, regenerative medicine, and bioremediation, underscoring their growing relevance across disciplines.

### 4.3. Bioengineering Approaches for Enhanced Exosome Production

Efficient and scalable production of cyanobacterial ELVs is crucial for therapeutic translation. Several bioengineering strategies are being explored to increase EV yield and functionality, drawing from microbial and mammalian systems.

Genetic engineering offers a targeted approach to enhancing vesicle biogenesis. Using genome-editing tools such as CRISPR-Cas (particularly Cpf1), researchers can overexpress genes associated with membrane curvature, vesicle budding, or lipid remodeling, or suppress pathways that limit vesicle release. In microbial systems, overexpression of outer membrane vesicle (OMV)-associated proteins or deletion of peptidoglycan-binding motifs has significantly improved vesicle yield, suggesting analogous strategies may be applicable to cyanobacteria [66].

Environmental and chemical stimuli such as oxidative stress, nutrient deprivation, or temperature shifts have also been shown to stimulate vesiculation in cyanobacteria. While cytokine treatments (e.g., IFN-γ or IL-1β) enhance exosome secretion in mammalian cells [67], equivalent signaling pathways in cyanobacteria remain largely undefined. Identifying stress-response regulators and engineering their upregulation may be a key strategy.

Three-dimensional (3D) culture systems, such as hydrogel scaffolds and microcarriers, mimic the in vivo extracellular matrix and enhance cell-cell communication. These platforms improve biomass density and metabolic output, resulting in increased vesicle yield. Applying such systems to cyanobacterial cultures could mimic biofilm conditions, facilitating enhanced ELV production [68].

Physical stimulation through shear stress, stretching, or pulsatile flow has also been used to induce EV secretion. For example, engineered tissue systems subjected to mechanical loading showed up to a 37-fold increase in EV release [69]. Adapting bioreactor-based agitation or fluid dynamics to cyanobacterial cultures could leverage mechanotransduction pathways for scalable production [70].

Collectively, these engineering strategies offer a roadmap to transform naturally limited EV output into biomanufacturing-compatible yields. However, specific regulatory circuits, membrane remodeling enzymes, and vesicle packaging mechanisms in cyanobacteria remain poorly understood and require dedicated mechanistic studies.

## 5. Potential of Cyanobacterial EVs in Drug Delivery

### 5.1. Biocompatibility and Stability of Cyanobacterial EVs

Cyanobacterial EVs exhibit high biocompatibility due to their natural origin and evolutionary compatibility with diverse biological systems. Derived from cyanobacteria ubiquitous, photosynthetic microorganisms that have coexisted with other life forms for over two billion years, these vesicles contain phospholipids, proteins, and nucleic acids that are generally well tolerated by mammalian cells [71]. Their endogenous-like composition reduces the likelihood of triggering strong immune responses, a feature that distinguishes them from many synthetic nanoparticles. Several studies have demonstrated that cyanobacterial EVs can interact with mammalian cell lines with minimal cytotoxicity and low immunogenicity, making them suitable for therapeutic applications [72,73]. For example, *Spirulina*-derived EVs have been characterized as biologically compatible in vivo and demonstrated utility as a vaccine adjuvant, with antigen-specific IgG responses increased by >100-fold compared with antigen alone in murine models, without overt toxicity [11]. In addition, *Synechococcus elongatus* PCC 7942 EVs promoted cutaneous wound repair in mice by enhancing angiogenesis and accelerating healing relative to controls, again without clear systemic safety signals in the reported model [55].

In addition to their immunological compatibility, cyanobacterial EVs demonstrate remarkable physical and chemical stability. Their lipid bilayer structure confers protection against enzymatic degradation, environmental stress, and mechanical shear, thereby preserving the integrity of encapsulated cargo [74]. Such durability is crucial for maintaining drug activity during systemic circulation. Furthermore, surface engineering techniques, including lipid modification and functional ligand conjugation, can further enhance vesicle stability and targeting potential [75]. Quantitative production and performance metrics from photosynthetic microorganism-derived EVs also support feasibility for translation: EV preparations from the microalga *Tetraselmis chuii* have been reported at high yields on the order of ~10^12^ particles·L^−1^ (approximately ~10^4^ EVs per cell), with good colloidal stability and in vivo biocompatibility, providing a useful benchmark for developing scalable cyanobacterial EV platforms [75]. From a comparative perspective, cyanobacterial EVs may complement established therapeutic EV platforms by offering potentially scalable, cultivation-based production, whereas mammalian exosomes, although more clinically advanced, remain constrained by donor variability, complex bioprocessing/QC (quality control), and stringent safety and regulatory requirements; synthetic nanocarriers (liposomes/polymeric nanoparticles) provide manufacturing control but can face stability and tolerability limitations depending on formulation [17,48,49]. Taken together, these attributes position cyanobacterial EVs as a promising class of natural nanocarriers with both biological safety and structural robustness for in vivo drug delivery.

### 5.2. Natural Loading of Bioactive Compounds in EVs

Cyanobacterial EVs are not only structurally competent as delivery vehicles but also demonstrate intrinsic ability to encapsulate bioactive compounds during vesicle formation. This natural loading process is highly selective and regulated, allowing cellular metabolites including proteins, lipids, pigments, and secondary metabolites to be selectively packaged into vesicles. These molecules can retain functional activity in recipient cells, modulating oxidative stress, inflammation, or gene expression [76]. As noted above, recent photosynthetic organism-derived EV studies provide useful translational benchmarks for scalable particle production and characterization workflows, which can inform optimization of cyanobacterial EV cargo profiling and formulation development [75].

This process is influenced by environmental cues and the physiological state of the cyanobacterial cells. For instance, stress-induced conditions such as nutrient limitation or oxidative stress can increase both EV secretion and the diversity of bioactive cargo. Marine cyanobacteria such as *Lyngbya majuscula* and *Symploca* spp. produce compounds like lagunamide A and largazole, which have shown potent anticancer activity via mitochondrial apoptosis and histone deacetylase inhibition, respectively [26]. The natural encapsulation of such compounds into ELVs suggests a built-in drug delivery system, evolutionarily optimized for chemical protection and delivery.

While the intrinsic loading mechanisms are efficient for endogenous metabolites, the encapsulation of exogenous therapeutic agents often requires augmentation. Most current loading strategies, such as passive diffusion or electroporation, have been developed and validated in mammalian or algal systems, and their direct applicability to cyanobacterial EVs remains to be experimentally confirmed. The unique lipid and protein composition of cyanobacterial membranes may influence loading efficiency and cargo compatibility.

Notably, EVs derived from related microalgae (e.g., *Tetraselmis chuii*) have demonstrated antioxidant, anti-inflammatory, and bone-targeting properties. While cyanobacteria and green algae are evolutionarily distinct, such findings suggest a promising direction for exploring similar properties in cyanobacterial vesicles, pending empirical validation. Additionally, their lipid membranes closely resemble those of mammalian cells, which reduces immunogenicity and improves circulation time [76,77].

### 5.3. Strategies for Drug Encapsulation and Targeted Delivery

To enhance the therapeutic utility of cyanobacterial EVs, several strategies have been proposed to improve both drug loading efficiency and targeted delivery. Co-incubation is a widely used, straightforward method for loading small molecules into pre-isolated exosomes. By exploiting concentration gradients, therapeutic agents, particularly hydrophobic compounds, can passively diffuse across the lipid bilayer. However, this approach is often less effective for hydrophilic molecules due to poor membrane permeability, resulting in low encapsulation efficiency and potential drug wastage [78,79].

Electroporation, commonly employed in mammalian exosome systems to load nucleic acids such as siRNA and miRNA, has not yet been systematically evaluated in cyanobacterial EVs. Given the differences in membrane composition and vesicle biogenesis between prokaryotic and eukaryotic systems, optimization of electrical parameters and thorough validation of vesicle integrity are essential before this method can be adapted to cyanobacteria-derived EVs [80].

Similarly, surfactant-based loading techniques, such as saponin-assisted membrane permeabilization, are well documented in synthetic and mammalian EVs. While theoretically applicable, their use in cyanobacterial systems remains speculative and requires careful assessment to avoid compromising vesicle stability or inducing cytotoxicity. Hybrid vesicle systems, formed by fusing liposomes with exosomes, offer enhanced flexibility for drug loading and targeting. These constructs are well-characterized in mammalian models and have demonstrated benefits such as improved stability, modular surface engineering, and increased drug payload [81]. However, analogous hybrid systems involving cyanobacterial EVs have not yet been reported in the literature. Nevertheless, this approach represents a promising frontier for future investigation.

Surface modification of exosomes through conjugation of targeting ligands such as peptides, antibodies, or aptamers has also been widely adopted to improve tissue-specific delivery in mammalian systems. Such strategies enhance cellular uptake and therapeutic index by leveraging ligand-receptor interactions at target sites [82]. Additionally, genetic engineering of the parent cyanobacteria offers an upstream and sustainable strategy for producing targeted EVs. By integrating sequences encoding targeting peptides or receptor-binding domains into the genome of exosome-producing cells, vesicles can be biosynthetically functionalized for selective delivery to tumors or inflamed tissues [83]. This approach has already been demonstrated in engineered mammalian and bacterial systems, though its application to cyanobacteria is still nascent.

It is important to acknowledge that many of the drug loading and targeting strategies discussed in this section are derived from studies in mammalian, algal, or bacterial systems. Their direct application to cyanobacterial EVs remains largely theoretical at this stage. Cyanobacteria differ substantially from eukaryotic cells in membrane architecture, lipid composition, and vesicle formation pathways. As such, experimental studies are needed to assess the feasibility, efficiency, and safety of these methods when applied to cyanobacterial EVs. Future research should prioritize the development of optimized protocols for vesicle purification, cargo retention, in vivo delivery performance, and immune response profiling, critical steps toward advancing the clinical translation of cyanobacterial EV -based drug delivery.

**Figure 2:**
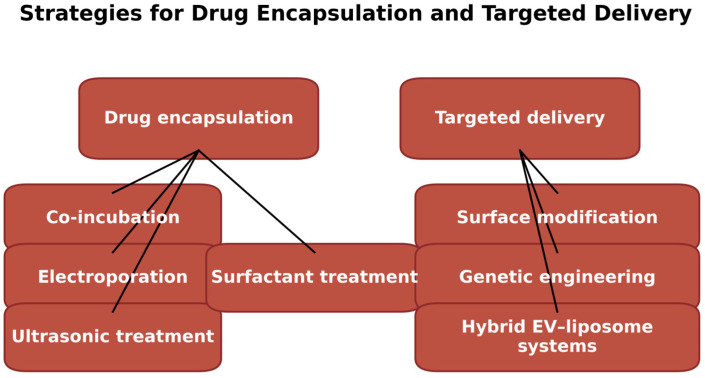
Schematic representation of drug encapsulation and targeting strategies using cyanobacterial EVs.

Figure 2 illustrates the diverse strategies applied for efficient drug encapsulation and targeted delivery using cyanobacterial EV-like vesicles. Drug loading can be achieved through physical techniques such as electroporation and ultrasonic treatment, which temporarily disrupt membrane integrity, or through chemical and biological approaches such as surfactant treatment and co-incubation that promote passive diffusion of therapeutic molecules. After encapsulation, delivery efficiency can be significantly enhanced through surface modification, enabling ligand-mediated targeting, or through genetic engineering to incorporate specific proteins or markers onto the vesicle surface. Additionally, hybrid exosome–liposome platforms offer a promising avenue for combining the natural biocompatibility of exosomes with the structural stability of liposomes. Together, these methods support the development of advanced vesicle-based drug delivery systems with improved therapeutic precision.

## 6. Cyanobacteria-Derived EVs in Therapeutics

While cyanobacteria have long been recognized as prolific producers of bioactive metabolites with diverse therapeutic properties, the exploration of their exosome-like EVs for clinical applications is still in its infancy. Nevertheless, the emerging field of EV/exosome-based drug delivery, well-studied in mammalian and algal systems, offers a compelling framework to evaluate the potential of cyanobacterial EVs. To enhance scientific depth, this section integrates quantitative benchmarks (e.g., potency metrics and in vivo performance readouts) where available, and explicitly indicates where data remain extrapolated from non-cyanobacterial systems. The following subsections highlight current knowledge and future possibilities across various therapeutic domains, noting where evidence is speculative and where cyanobacterial contributions are directly supported.

### 6.1. Applications in Anti-Cancer Therapies

Cyanobacteria produce a wide array of cytotoxic compounds with demonstrated anticancer activity. Among these, dolastatins linear peptides derived from *Symploca* species have shown potent microtubule-targeting effects, leading to mitotic arrest and apoptosis. Notably, dolastatin-10 analogs such as brentuximab vedotin have been developed as antibody–drug conjugates and approved for Hodgkin lymphoma and anaplastic large cell lymphoma by the U.S. FDA (Food and Drug Administration) [84]. Similarly, curacin A, isolated from *Lyngbya majuscula*, binds the colchicine site of tubulin, disrupting microtubule polymerization and promoting apoptosis [85]. Beyond these exemplars, multiple marine cyanobacterial metabolites display high in vitro potency, often in the nanomolar-to-low micromolar range (IC_50_), underscoring their suitability as payload candidates for vesicle-based delivery systems [37,85].

Although the anticancer potential of these metabolites is well established, the use of cyanobacterial EVs as delivery vehicles for such agents remains largely unexplored. In mammalian systems, exosome-based carriers have demonstrated measurable efficacy improvements in tumor models, including enhanced tumor accumulation and improved therapeutic indices relative to free drug, while minimizing systemic toxicity [86]. Incorporating cyanobacterial-derived compounds into EVs could enhance their therapeutic index by improving stability, solubility, and selective uptake by cancer cells [87]. A practical performance benchmark for future cyanobacterial EV studies is the magnitude of tumor-growth suppression observed with engineered exosome systems in vivo (e.g., tumor volume reductions on the order of ~30–50% in representative models depending on cargo and platform), alongside biodistribution and tolerability endpoints [86,87]. However, this concept remains hypothetical in the context of cyanobacteria, and targeted experimental research is required to assess vesicle compatibility, tumor targeting, and biodistribution. In summary, cyanobacterial bioactives have a strong foundation in oncology, and leveraging EVs for their delivery represents an exciting yet underexplored avenue.

### 6.2. Use in Antimicrobial and Antiviral Treatments

#### 6.2.1. Antimicrobial Applications

Cyanobacterial metabolites such as berberine and bacteriocin-like peptides exhibit potent antimicrobial activities via multiple mechanisms, including quorum sensing inhibition, membrane disruption, and pathway interference [27,88]. Where reported, antimicrobial peptide activity is often quantified by minimum inhibitory concentrations (MICs) and membrane-disruption readouts, providing clear efficacy metrics for prioritizing cargo candidates [88,89]. Encapsulation of these compounds within exosomes may protect them from enzymatic degradation and improve their pharmacokinetic properties, as has been shown in mammalian systems [89]. While such encapsulation strategies are not yet demonstrated in cyanobacterial vesicles, their potential to reduce antimicrobial resistance through targeted, low-dose delivery remains a promising research direction. Future cyanobacterial EV studies could adopt quantitative benchmarks used in antimicrobial nanocarrier testing (e.g., MIC shifts, time–kill kinetics, and biofilm disruption percentages) to objectively compare EV formulations against free-drug controls and polymeric/liposomal carriers [6,7,17,27,38,49,89].

#### 6.2.2. Antiviral Applications

Cyanobacteria also produce antiviral compounds with mechanisms that interfere with viral entry, replication, or immune evasion. Lectins such as cyanovirin-N (from *Nostoc ellipsosporum*), scytovirin (*Scytonema varium*), and microvirin (*Microcystis aeruginosa*) have shown activity against HIV and other enveloped viruses by binding viral envelope glycoproteins [84,90,91]. Additionally, polysaccharides like calcium spirulan from *Spirulina platensis* block viral fusion and replication in HIV, herpes simplex virus, and human cytomegalovirus [92]. Several of these antiviral agents have been evaluated with quantitative virological endpoints (e.g., EC_50_/IC_50_ values, viral-load reductions, and inhibition of viral entry/fusion), which can serve as payload-selection metrics for EV-based delivery strategies [84,90,91,92]. In other systems, exosome-mediated antiviral delivery has successfully delivered antiviral agents to infected cells with high specificity, prolonging circulation and minimizing toxicity [93]. If cyanobacterial EVs demonstrate similar delivery capabilities, they may serve as next-generation nanocarriers for antiviral therapies though direct validation in cyanobacteria remains pending.

### 6.3. Potential in Neuroprotective and Regenerative Medicine

Cyanobacterial compounds possess neuroprotective properties primarily attributed to their antioxidant and anti-inflammatory effects. These activities target key pathological pathways in neurodegenerative diseases such as Alzheimer’s and Parkinson’s [94]. While research on cyanobacterial EVs in the nervous system is still lacking, analogies from other systems suggest substantial therapeutic potential.

For instance, EVs/exosomes from neural stem cells and microalgae have shown promise in enhancing neuronal survival, reducing inflammation, and promoting regeneration in models of stroke, multiple sclerosis, and spinal cord injury [95]. In particular, engineered exosomes loaded with neuroprotective agents like quercetin have demonstrated efficacy in cerebral ischemia models [96]. Importantly, such studies typically report quantitative outcome measures (e.g., infarct volume, neurological deficit scores, inflammatory cytokine panels), providing performance models that future cyanobacterial EV studies can adopt for rigorous evaluation [95,96]. Moreover, EVs derived from *Spirulina* platensis integrated with herbal hydrogels have improved chondrocyte survival and restored mitochondrial function in osteoarthritis models [97]. These examples illustrate the feasibility of vesicle-based neurotherapy and support the rationale for exploring similar strategies in cyanobacteria-derived EVs. In parallel, cyanobacterial antioxidants such as C-phycocyanin have demonstrated neuroprotective and anti-inflammatory effects in established experimental contexts, which can be quantified via oxidative-stress markers and inflammatory mediators when translated into EV-based delivery designs [31,98].

### 6.4. Role in Modulating Immune Responses and Inflammation

Cyanobacteria also generate bioactive components capable of modulating immune responses. One study reported that exopolysaccharides from *Cyanobacterium aponinum* induced a tolerogenic dendritic cell phenotype, increasing IL-10 secretion and promoting regulatory T cell differentiation while suppressing pro-inflammatory Th17 responses [99]. Although not directly linked to EVs, this immunomodulatory effect suggests that EV-associated delivery may enhance or refine immune targeting. Notably, *Spirulina*-derived EVs have also been evaluated as vaccine adjuvants with quantitative immune readouts (e.g., >100-fold increases in antigen-specific IgG titres compared with antigen alone), supporting the feasibility of EV-associated immune modulation with measurable endpoints [11].

Supporting this hypothesis, exosome-mediated immune modulation in mammalian systems has been shown to shift macrophage polarization toward an anti-inflammatory phenotype via the SOCS3/JAK2/STAT3 axis, thereby improving outcomes in spinal cord injury models [100]. While such findings are not yet replicated in cyanobacterial systems, they underline the broader potential of EV based immune modulation. For future cyanobacterial EV translation, quantitative immunogenicity/safety benchmarking should include cytokine profiling (e.g., IL-6, TNF-α, IL-10), complement activation panels, and anti-EV antibody responses, consistent with current guidance for EV therapeutics development [18,101].

As summarized in Table 4, cyanobacteria-derived compounds and vesicles show mechanistic relevance across diverse therapeutic domains, including oncology, infectious disease, neurology, regenerative medicine, and immunology. Compounds such as dolastatins and curacin A exhibit potent anticancer activity, while antiviral agents like cyanovirin-N and calcium spirulan target viral entry and replication. Antimicrobial peptides, antioxidants, and immunomodulatory polysaccharides further contribute to their pharmacological repertoire.

EV-mediated delivery of these agents may enhance their stability, targeting precision, and therapeutic efficacy. Although direct experimental validation of cyanobacterial EV-based applications is still limited, parallel findings from mammalian and algal systems strongly suggest translational potential. To strengthen scientific evaluability, future studies should report standardized quantitative metrics, particle size/distribution, zeta potential, yield (particles·L^−1^), cargo loading efficiency (%), release kinetics, and biodistribution together with domain-specific efficacy readouts (e.g., IC_50_/EC_50_, viral-load reductions, MIC shifts, wound-closure rates, histological scoring, and cytokine fold-changes), enabling direct comparison against liposomal, polymeric, and mammalian exosome benchmarks. Future studies should focus on optimizing drug-loading strategies, evaluating in vivo delivery performance, and characterizing immune responses to fully harness the therapeutic promise of cyanobacterial EVs.

## 7. Challenges and Future Directions

### 7.1. Limitations in Isolating and Characterizing Cyanobacterial EVs

The isolation and characterization of EVs from cyanobacteria present unique technical challenges that currently limit their application in drug delivery. One major obstacle is the complex extracellular environment of cyanobacteria, which is rich in polysaccharides and proteins that tend to co-purify with EVs, leading to contamination that hinders downstream analyses [117]. For example, extracellular carbohydrate polymers can obscure low-abundance proteins, compromising proteomic and RNA-based profiling [117,118].

A further challenge arises from the heterogeneity of vesicle populations. Cyanobacteria release diverse types of EVs that differ in size, composition, and function, making it difficult to isolate EV subpopulations with high purity. Contaminants such as cell debris and extracellular polymeric substances further complicate the isolation process and reduce reproducibility [118].

Although methods such as ultracentrifugation and size exclusion chromatography (SEC) are widely used for mammalian exosomes/EVs, they remain suboptimal for cyanobacterial systems. The high viscosity of cyanobacterial cultures impairs sedimentation efficiency and requires protocol adjustments [119]. While SEC has shown promise for microbial vesicle purification, its performance in cyanobacterial applications still needs validation and optimization to improve yield and purity [54,117].

Emerging approaches, including tangential flow filtration, affinity capture, and microfluidics, offer promising alternatives and warrant investigation in cyanobacterial contexts [120]. Developing standardized, species-specific protocols and combining multiple complementary techniques may enhance yield, purity, and downstream functional characterization. Notably, recent studies on microalgae-derived EVs (“nanoalgosomes”) have begun to standardize isolation/QC workflows and report reproducible characterization outputs (e.g., size distribution, particle counts, and functional assays), providing practical methodological benchmarks that could be adapted to cyanobacterial systems [12,75].

**Figure 3:**
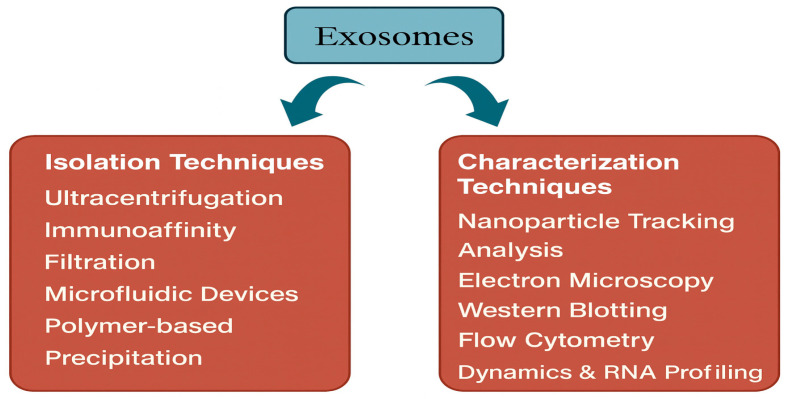
Techniques for Isolating and Characterizing Cyanobacterial EVs. Schematic overview of current and emerging strategies adapted for EVs purification from cyanobacteria, including ultracentrifugation, size exclusion chromatography, filtration, and microfluidic separation.

Figure 3 provides a comprehensive overview of the major techniques utilized for isolating and characterizing exosome-like vesicles/EVs derived from cyanobacteria. Differential ultracentrifugation remains the most widely used method, offering efficient sedimentation of vesicles based on density. Complementary techniques such as size exclusion chromatography and membrane filtration further refine vesicle purity by removing protein aggregates and cellular debris. In recent years, microfluidic platforms have emerged as innovative tools that enable rapid, high-resolution separation of nanoscale vesicles with minimal sample loss. Together, these strategies form an integrated framework for obtaining high-quality cyanobacterial EVs suitable for biochemical, molecular, and therapeutic applications.

### 7.2. Scale-Up Challenges for Industrial Applications

Translating laboratory-scale cyanobacterial EVs production to industrial-scale systems involves several significant challenges. A primary bottleneck is optimizing culture conditions cyanobacteria are highly sensitive to environmental variables such as light intensity, temperature, pH, and nutrient levels. Maintaining consistency across large scale photobioreactors poses difficulties that can impact both biomass yield and EV production [121].

Bioreactor design adds further complexity. Traditional systems often suffer from limitations in light penetration, gas exchange, and mixing efficiency. These issues compromise cell density and EV output. Scalable photobioreactor designs must address such limitations to achieve uniform and productive growth [122].

The downstream isolation and purification of EVs from large volumes of culture media also remains inefficient. Standard techniques like ultracentrifugation, ultrafiltration, and SEC are difficult to scale and often lack throughput [69,123]. New approaches, such as high-capacity tangential flow systems or chromatography-based automation are urgently needed for industrial feasibility.

Quality control is essential to ensure batch-to-batch consistency in EV yield and content. Even minor variations in membrane composition or cargo can affect therapeutic efficacy and safety [122,124]. Establishing robust characterization protocols, including particle size distribution, surface markers, and bioactive cargo will be key for clinical translation.

Finally, EV-based therapeutics must meet rigorous regulatory standards, including compliance with current Good Manufacturing Practices (cGMP). Production processes will require strict documentation, reproducibility, and contamination control to satisfy global regulatory expectations [125,126]. Importantly, the rapid maturation of microalgae-derived EV platforms provides a useful translational analogue: recent work has demonstrated that photosynthetic organism-derived EVs can be produced at high particle numbers and evaluated in vivo for biodistribution and therapeutic performance, offering concrete “scale-up and validation” models that cyanobacterial EV manufacturing efforts can benchmark against [12,75]. Addressing these scale-up challenges will require collaborative efforts between synthetic biologists, engineers, and regulatory scientists.

### 7.3. Need for Regulatory and Safety Assessments

The successful clinical translation of cyanobacteria-derived EVs hinges on rigorous regulatory and safety evaluations. Due to their biological origin and complex structure, EV therapeutics (including exosome-based products) are often classified as biologics or advanced therapy medicinal products (ATMPs), requiring comprehensive data on molecular composition, manufacturing reproducibility, pharmacokinetics, and therapeutic efficacy [127,128].

A lack of harmonized international standards adds complexity to the approval process. Differences in isolation techniques, vesicle heterogeneity, and purification methods can affect consistency across production batches [129]. Regulatory bodies are still developing frameworks to evaluate these novel biologics, posing a challenge for emerging technologies like cyanobacterial EVs.

Safety assessments are particularly critical. Contaminants from source cultures, such as endotoxin-like components, residual toxins or extracellular polymeric substances, must be removed through validated purification steps to prevent inflammatory or toxic effects [80,101]. Furthermore, exosomes must retain integrity and functionality during storage and transport. Stability testing under clinical conditions is essential to prevent loss of cargo or activity [130].

Although EVs/exosomes are generally considered to exhibit low immunogenicity, it remains important to evaluate their biodistribution, clearance, and potential off-target effects through in vivo pharmacokinetic studies [130,131]. Preclinical toxicology, long-term safety studies, and immunological profiling will be indispensable in building a foundation for human application. Recent microalgae-derived EV studies that include in vivo biodistribution and functional endpoints highlight the feasibility of performing such safety and performance benchmarking in photosynthetic EV platforms, and they reinforce the value of harmonized QC and reporting standards for cyanobacterial EV translation [75].

### 7.4. Future Biotechnological Advancements and Synthetic Biology Integration

The integration of synthetic biology and nanobiotechnology is poised to transform cyanobacterial EVs into programmable and highly functional drug delivery systems. Cyanobacteria can be genetically modified to enhance vesicle biogenesis, load specific therapeutic cargo, and display targeting ligands on the EV membrane, thereby improving specificity and efficacy [75,132].

Beyond engineering endogenous vesicles, synthetic exosomes constructed via charge-mediated assembly or lipid templating offer control over vesicle size, stability, and cargo encapsulation. These artificial constructs can mimic natural EV/exosome function while overcoming issues such as low yield or cargo leakage [133].

Additionally, bioinspired exosome-like nanoparticles have emerged as an alternative strategy. These hybrid systems combine the natural bioactivity of EV/exosomal components with synthetic modularity, offering advantages in cargo stability, targeted delivery, and scalable manufacturing [134].

Advances in genome editing, metabolic pathway reprogramming, and microfluidic bioreactor design will support this shift toward highly tunable vesicle systems. Collectively, these technologies promise to overcome current limitations and enable the development of next-generation EV based platforms for clinical use. In this context, recent microalgae EV advances, particularly reproducible characterization workflows, scalable production, and proof-of-concept in vivo applications, provide a near-term blueprint for accelerating cyanobacterial EV engineering and translation, while preserving the distinct advantages of cyanobacterial metabolism and genetic tractability [12,65,75,82].

## 8. Conclusions

Cyanobacteria represent a promising and underutilized source of bioactive compounds and naturally derived vesicles with potential applications in drug delivery. Their ability to produce exosome-like EVs that are biocompatible, stable, and capable of encapsulating diverse therapeutic cargos positions them as valuable candidates for next-generation delivery systems. These vesicles offer a unique intersection between natural product pharmacology and targeted delivery, with plausible applications across cancer therapy, infectious diseases, neurodegenerative disorders, and immune modulation.

While substantial promise exists, current evidence remains largely preliminary, and several critical barriers must be addressed. Technical challenges in vesicle isolation and characterization, scale-up limitations, and regulatory uncertainties must be systematically resolved. Advances in omics profiling, vesicle engineering, and controlled bioprocessing will be instrumental in overcoming these hurdles.

Future research should prioritize the development of efficient purification protocols, detailed characterization of vesicle composition, and preclinical evaluation of biodistribution, immunogenicity, and therapeutic performance. Genetic modification of cyanobacteria to tailor vesicle content or surface markers could significantly improve targeting and functional delivery. Furthermore, expanding the potential of cyanobacterial EVs beyond medicine to include environmental or agricultural applications offers exciting opportunities for interdisciplinary innovation.

By integrating the natural biosynthetic potential of cyanobacteria with the precision and flexibility of modern bioengineering, it may be possible to develop highly customized, scalable, and safe delivery platforms. This fusion of natural product science with nanotechnology could unlock novel therapeutic strategies and help overcome limitations of conventional drug delivery systems

## Figures and Tables

**Table 1:** Representative Cyanobacterial Metabolites with Therapeutic Potential and Prospects for Exosome-Based Delivery.

Metabolite/Class	Cyanobacterial Source	Pharmacological Activity	Mechanism of Action	Exosome Delivery Potential	References
Phycocyanin	*Spirulina* (*Arthrospira*)	Antioxidant, Anti-inflammatory, Anticancer	ROS scavenging, BCl-2 inhibition, Caspase-3 activation	High—hydrophilic, biocompatible, immune-tolerant	[31]
Microcystins	*Microcystis* spp.	Anticancer (toxic)	Inhibits protein phosphatases (PP1, PP2A)	Moderate—requires detox modification	[32]
Curacin A	*Lyngbya majuscula*	Antimitotic, Anticancer	Binds tubulin, inhibits microtubule polymerization	High—small molecule with cytoplasmic targets	[33]
Apratoxins	*Moorea* spp.	Cytotoxic, Antitumor	Disrupts cotranslational translocation, protein synthesis	High—potent, but toxicity needs modulation	[34]
Anatoxin-a/Saxitoxin	*Anabaena*, *Aphanizomenon*, *Cylindrospermopsis*	Neurotoxic (investigational for CNS drugs)	Blocks voltage-gated sodium channels	Low—potential risks, narrow therapeutic window	[35]
Berberine (cyanobacterial origin)	Reported in *Oscillatoria* spp.	Antimicrobial, anti-biofilm	Quorum sensing inhibition, membrane disruption	Moderate—may enhance efficacy via targeted delivery	[36]
Lagunamide A	*Lyngbya majuscula*	Anticancer	Mitochondria-mediated apoptosis	High—well suited for nanoparticle encapsulation	[37]

**Table 3:** Key Cyanobacterial Species Producing EVs, Their Functional Roles, and Potential Applications.

Cyanobacterial Species	EV Composition/Features	Biological Role/Mechanism	Potential Applications	References
*Prochlorococcus* (MIT9312/9313)	Lipids, proteins, DNA fragments	Nutrient exchange, microbial interactions, organic matter cycling	Marine biotechnology, environmental drug delivery	[53]
*Synechococcus elongatus* PCC7942	EVs enriched in pro-angiogenic factors	Promotes endothelial cell proliferation, enhances angiogenesis in wound healing	Regenerative medicine, wound repair therapies	[56]
*Synechocystis* sp. PCC 6803	Outer membrane proteins, periplasmic enzymes, metabolites	Stress adaptation, toxin removal, oxidative stress defense	EV-based drug carriers, stress biomarkers	[63]
*Cylindrospermopsis raciborskii*	Proteins, lipids, possible toxins	Microbial community signaling, toxin transport	Bioremediation, environmental biosensing	[64]
*Anabaena* sp. PCC 7120	Presumed protein and enzyme content (e.g., nitrogenase)	Intra-filament signaling, nitrogen metabolism during environmental stress	Biofertilizer enhancement, microbial ecology tools	[65]
*Spirulina* (*Arthrospira platensis*)	Nutritional and bioactive molecules (e.g., polysaccharides, proteins)	Immunomodulation, anti-inflammatory signaling, intercellular communication	Functional foods, vaccine adjuvants, nutraceuticals	[11]

**Table 4:** Therapeutic Applications of Cyanobacteria-Derived EVs and Bioactive Compounds.

Therapeutic Area	Bioactive Compound/Vesicle	Mechanism of Action	Exosome-Based Potential	Evidence Status	References
Cancer	Dolastatins (*Symploca* spp.), Curacin A (*Lyngbya majuscula*)	Microtubule inhibition, apoptosis induction	Exosomal delivery may enhance tumor targeting and reduce systemic toxicity	Direct in cyanobacteria: Supported in mammalian systems	[102,103,104]
Antimicrobial	Berberine, peptides, lipid disruptors	Quorum sensing inhibition, membrane disruption, pathway interference	Encapsulation could improve stability and reduce resistance	Direct in cyanobacteria: Conceptual only	[105,106,107]
Antiviral	Cyanovirin-N, Scytovirin, Calcium spirulan	Envelope glycoprotein binding, replication inhibition	Targeted antiviral delivery and immune shielding via exosomes	Direct in cyanobacteria: Demonstrated in microalgae & mammals	[108,109,110,111]
Neuroprotection	Antioxidants, anti-inflammatory compounds	ROS scavenging, inflammation suppression	Exosomal delivery may improve BBB penetration and neuron targeting	Direct in cyanobacteria: Parallels in *Spirulina* and stem cells	[98,112]
Regenerative Medicine	*Spirulina*-derived EVs	Mitochondrial protection, cartilage regeneration	Potential for tissue repair and osteoarthritis treatment	Indirect in cyanobacteria: (*Spirulina*)	[113,114]
Immunomodulation	Exopolysaccharides (*Cyanobacterium aponinum*)	Regulatory DC induction, IL-10 secretion, Th17 suppression	Exosomal enhancement of immune modulation	Direct in cyanobacteria: Partial Requires further vesicle-based validation.	[115,116]

## Data Availability

No new data were created or analyzed in this study. Data sharing is not applicable to this article.

## Abbreviations

3D	Three-Dimensional
ATMP	Advanced Therapy Medicinal Product
BCl-2	B-cell Lymphoma 2 (anti-apoptotic protein)
BDNF	Brain-Derived Neurotrophic Factor
BBB	Blood–Brain Barrier
CBB	Calvin–Benson–Bassham
CD9/CD63/CD81	Cluster of Differentiation 9/63/81 (tetraspanins)
CNS	Central Nervous System
Cpf1	CRISPR from *Prevotella* and *Francisella* 1 (a CRISPR-associated nuclease)
CRISPR-Cas	Clustered Regularly Interspaced Short Palindromic Repeats–CRISPR-associated proteins
CYP-450	Cytochrome P-450 system
DC	Dendritic Cell
DNA	Deoxyribonucleic Acid
ELVs	Exosome-Like Vesicles
ESCRT	Endosomal Sorting Complex Required for Transport
EVs	Extracellular Vesicles
FDA	Food and Drug Administration
HIV	Human Immunodeficiency Virus
Hsp70	Heat Shock Protein 70
Hsp90	Heat Shock Protein 90
HMECs	Human Microvascular Endothelial Cells
IFN-γ	Interferon-gamma
IL-1β	Interleukin-1 beta
ILVs	Intraluminal Vesicles
JAK2	Janus Kinase 2
lncRNA	Long Non-Coding RNA
MHC	Major Histocompatibility Complex
miRNA	MicroRNA
mRNA	Messenger Ribonucleic Acid
MVBs	Multivesicular Bodies
NDF	Neurotrophic Differentiation Factor
OMV	Outer Membrane Vesicle
PLGA	Poly (lactic-co-glycolic acid)
PP1/PP2A	Protein Phosphatase 1/Protein Phosphatase 2A
QC	Quality Control
ROS	Reactive Oxygen Species
SEC	Size Exclusion Chromatography
SOCS3	Suppressor of Cytokine Signaling 3
STAT3	Signal Transducer and Activator of Transcription 3
TSG101	Tumor Susceptibility Gene 101
